# Supplementary material for: Action of Multiple Rice β-Glucosidases on Abscisic Acid Glucose Ester
Source: Int J Mol Sci. 2021 Jul 15;22(14):7593. doi: 10.3390/ijms22147593 (PMC8303963; doi:10.3390/ijms22147593)

Supplementary data

Action of multiple rice β-glucosidases on abscisic acid glucose ester

Manatchanok Kongdin[1], Bancha Mahong[2], Sang- Kyu Lee[2], Su- Hyeon Shim[2], Jong-Seong Jeon[2,*], and James R. Ketudat Cairns[1,*]

[1] School of Chemistry, Institute of Science, Center for Biomolecular Structure, Function and Application, Suranaree University of Technology, Nakhon Ratchasima 30000, Thailand

[2] Graduate School of Biotechnology and Crop Biotech Institute, Kyung Hee University, Yongin 17104, Korea

* Correspondences: JJ, jjeon@khu.ac.kr, Tel.: +82 31 2012025 and JKC, cairns@sut.ac.th, Tel: +66 44 224304, Fax: +66 44 224185

Supplementary Table S1. PCR primers used in this study.

Supplementary Figure S1 Subcellular localization of Os4BGlu10-GFP and Os4BGlu13-GFP in coleoptile cells of transgenic rice plants

Supplementary Figure S2 Root and shoot lengths of seedlings of wild type Arabidopsis and transgenic lines expressing rice β-glucosidases under control conditions.

Supplementary Figure S3. Sodium dodecyl sulfate polyacrylamide gel electrophoresis analysis of *E. coli*-expressed Os4BGlu12 and Os4BGlu13 purified by IMAC and cut with enterokinase.

Supplementary Table S1. PCR primers used in this study.

Primer name	Sequence (5'→3')
Os4BGlu9 cloning F	5′-CACCGAATTGCAGGTCAAGCTTCGT-3′
Os4BGlu9 cloning R	5′-ACACATTGGGATTAAGTGCCAA-3′
Os4BGlu10 cloning F	5′-CACCCACTCTTTTCTGTCTATGTAGG -3′
Os4BGlu10 cloning R	5′-CATGCAAGAAGAGGAGAGTGAG-3′
Os4BGlu11 cloning F	5′-TCTTCTGTCTATGTAGGTCATG-3′
Os4BGlu11cloning R	5′-CTTATATACTATTTCGATGTTC-3′
Os4BGlu12 cloning F	5′-GTTGATGGCACCAAACATTG-3′
Os4BGlu12 cloning R	5′-AAGCAATGAGTGCAATGGTGC-3′
Os4BGlu13 cloning F	5′-CACCGCGGGTCACACACACATACT3′
Os4BGlu13 cloning R	5′-GGGAAGAGCAATGTGTGCAACT-3′
GFPBGlu9F	5′-CACCATGGCGGTTGCCGGGGCAGTGGCGA-3′
GFPBGlu9R	5′-ATGTCTGTACTGGCAAACTCT-3′
GFPBGlu10F	5′-CACCATGGCGGTTGCAGGTGCAATGGTGAT-3′
GFPBGlu10R	5′-TTTCCGGAGGAACTTCTTGAACCA-3′
GFPBGlu11F	5′-CACCATGGCGGTTGCAGGGGCAATGGT-3′
GFPBglu11R	5′-ATTAGAAAAGGCATTGTATGCA-3′
GFPBGlu12F	5′-CACCATGGCAGATGGAAGTCTGAGGGGTGG-3′
GFPBGlu12R	5′-TTTCAGGAGGAACTTCTTGAACCAAT-3′
GFPBGlu13F	5′-CACCATGGCAGCTGCAGGGGAAGTGGTGA-3′
GFPBGlu13R	5′-TTTCTGGAGGAACTCCTTGAACC-3′
RTBGlu9F	5′-GCACTGGAAGATAAATACAACGGATT-3′
RTBGlu9R	5′-TGATATTTCTCTCTGTACAACCGAA-3′
RTBGlu10F	5′-TGAAATCAATAATAAGACCATGCGAC-3′
RTBGlu10R	5′-TCATTTCCGGAGGAACTTCTTGAACCA-3′
RTBGlu11F	5′-CACCTCTTCTGTCTATGTAGGTCATG -3′
RTBGlu11R	5′-TCAATTAGAAAAGGCATTGTATGCAAC-3′
RTBGlu12F	5′-CACCGTTGATGGCACCAAACATTG-3′
RTBGlu12R	5′-TCATTTCAGGAGGAACTTCTTGAACCAAT-3′
RTBGlu13F	5′-TGAATTCAACAATAAGACCTTACCACT-3′
RTBGlu13R	5′-TCATTTCTGGAGGAACTCCTTGAACC-3′

Supplementary Figure S1 Subcellular localization of Os4BGlu10-GFP and Os4BGlu13-GFP in coleoptile cells of transgenic rice plants. (A-H) Coleoptile cells of Os4BGlu10-GFP (A-D) and Os4BGlu13-GFP (E-H). A and E, GFP fluorescence. B and F, FM4-64 red signal. C and G, Bright field. D and H, Merged. (I-P) Plasmolysed coleoptile cells of Os4BGlu10-GFP (I-L) and Os4BGlu13-GFP (M-P). I and M, GFP fluorescence. J and N, FM4-64 red signal. K and O, Bright field. L and P, Merged.

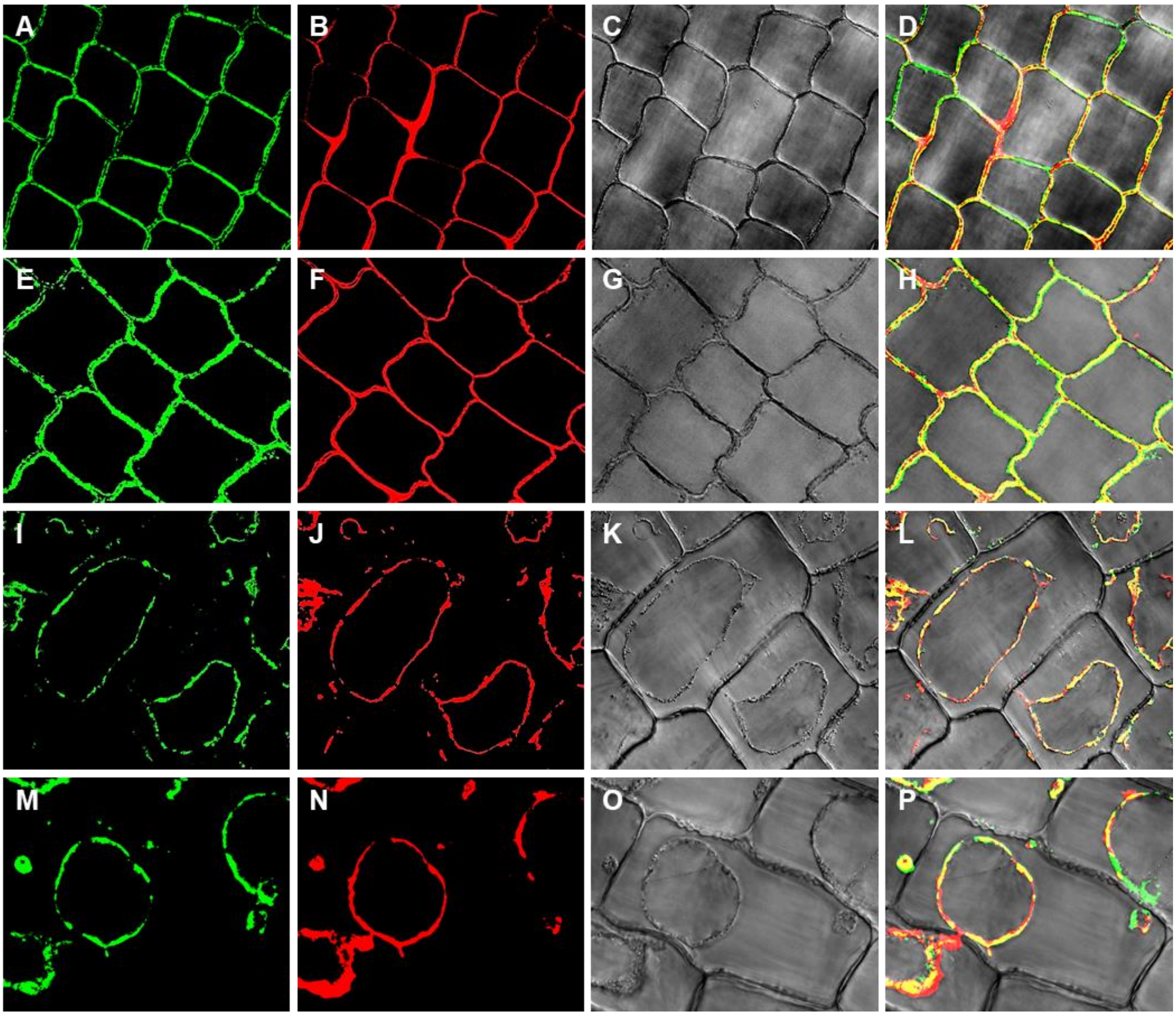

Supplementary Figure S2 Root and shoot lengths of seedlings of control Arabidopsis and transgenic lines expressing rice β-glucosidases under control conditions. Control and transgenic Arabidopsis expressing rice phytohormone β-glucosidases Os4BGlu9-13 were grown on ½ MS plates for 7 d and transplanted to ½ MS for 5 d. To quantify root and shoot growth inhibition, root and shoot lengths were measured in three independent experiments with 20 plants each

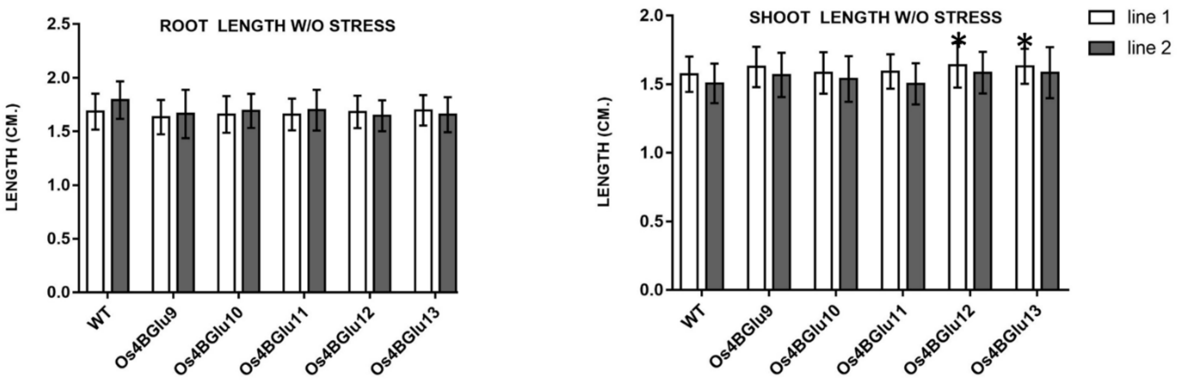

Supplementary Figure S3. Sodium dodecyl sulfate polyacrylamide gel electrophoresis analysis of *E. coli*-expressed Os4BGlu12 and Os4BGlu13 purified by IMAC and cut with enterokinase. Lane 1: Low Molecular Weight Protein Marker (GE Healthcare); Lane 2: Os4BGlu12; and Lane 3: Os4BGlu13.

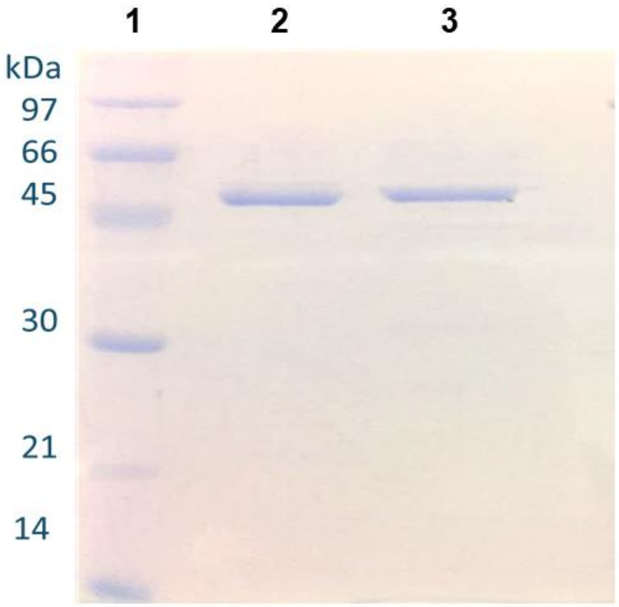

Supplement: Supplementary file 1 [file ijms-22-07593-s001.zip › ijms-1210391-supplementary.pdf]
